# Green synthesis of spermine coated iron nanoparticles and its effect on biochemical properties of *Rosmarinus officinalis*

**DOI:** 10.1038/s41598-023-27844-5

**Published:** 2023-01-14

**Authors:** Mehdi Afrouz, Farnaz Ahmadi-Nouraldinvand, Sabry G. Elias, Mohammad Taghi Alebrahim, Te Ming Tseng, Hoda Zahedian

**Affiliations:** 1 Department of Plant Production and Genetics, University of Mohaghegh Ardabili, Ardabil, Iran; 2 Department of Crop and Soil Science, Oregon State University, Corvallis, USA; 3 Department of Plant and Soil Science, Mississippi State University, Starkville, USA; 4 Department of Deutsch-Sprachen, Volkshochschule, Gütersloh, Germany

**Keywords:** Biochemistry, Biological techniques, Biotechnology, Chemical biology, Plant sciences, Materials science

## Abstract

In this study, aqueous spinach extract was used for the green synthesis of iron nanoparticles. The surface of iron oxide nanoparticles was coated with spermine. The physicochemical properties of nanoparticles were investigated using UV-Vis, TGA, FTIR, VSM, TEM, and DLS. The results showed that the nanoparticles had a spherical structure. The surface charge of the Fe_3_O_4_-NPs increased from −3.2 to 18.42 (mV) after Fe_3_O_4_ coating by spermine. In order to investigate the effect of nanoparticles on physicochemical properties of rosemary under drought stress conditions, an experiment was carried out in a completely randomized design. The results showed that the amount of antioxidant enzymes and secondary metabolites increased significantly under drought stress. Moreover, the use of spermine-coated iron nanoparticles can be useful in increasing resistance to drought stress in plants by increasing the activity of some antioxidant enzymes and secondary metabolites. The biocompatibility of Nanoparticles in cell suspension was investigated. the ability of Fe_3_O_4_-SM NPs to interact with DNA and protect it against DNaseI and ultrasonic waves using agarose gel electrophoresis was studied. The ability of Fe_3_O_4_-SM to neutralize the negative charge of DNA and protect it against DNaseΙ and ultrasonic waves was confirmed using an agarose gel electrophoresis assay.

## Introduction

Currently, the lack of water (drought stress) on farms is one of the most critical issues in the agricultural sector. Drought stress hurts plant growth and productivity by causing physiological, biochemical, and molecular changes within the plant. It is one of the most severe abiotic stresses limiting plant growth and yield worldwide^1,2^. As a result, using some nanoparticles in drought conditions is one of the most effective ways to reduce the harmful effects of drought stress on plants. In recent decades, nanotechnology has made tremendous progress in various fields, such as agriculture, medicine, and industry. The use of different nanoparticles is increasing rapidly in multiple areas due to their unique properties. One of the elements used in the nanoparticles is iron. Iron is involved in a variety of biochemical and physiological processes and serves as a co-factor for several enzymes that act as catalysts in a variety of biochemical reactions^3^. Iron oxide nanoparticles (Fe_3_O_4_-NPs) are also increasing in popularity due to their potential applications in environmental remediation and biomedicine^4^. The green synthesis of nanoparticles using plant compounds is a new method to synthesize various nanoparticles. In other words, plant extract nanoparticle synthesis is an extracellular process in which plant extracts are directly used to synthesize nanoparticles^5^. This method is more popular than chemical methods due to its good biocompatibility, and low production costs^6,7^. In a way, green Fe_3_O_2_-NPs synthesis has become a better choice for scientists today when compared to other synthesis methods because the synthesis techniques are non-toxic and environmentally friendly^8^. Researchers have reported that under drought stress, iron oxide nanoparticles synthesized from ginger and cumin have been reported to increase proline, superoxide dismutase, peroxidase, and ascorbate peroxidase in wheat^9^.

The use of microorganisms to synthesize nanoparticles is another method with high bio-compatibility to protect nanomaterials. However, the use of living organisms to prepare metal nanoparticles is expensive and limited. Plants are considered potential sources for the production of nanomaterials because of their widespread availability and low cost^10,11^. In addition, plant extracts are a good alternative for use as a catalyst in synthesizing metal nanoparticles^12^. Interestingly, previous research has shown that nanoparticles synthesized based on the green synthesis method have a greater ability to be absorbed into the cells than those synthesized using chemical methods^13^. This may be due to various proteins, fibers, and carbohydrates on the surface of nanoparticles synthesized using plant compounds. Therefore, the uptake of these nanoparticles increases through the proteins, fibers, and carbohydrate receptors on the cell surface^14,15^.

Rosemary, which is also known by the scientific name *Rosmarinus officinalis L* is a medicinal plant of the mint family. Rosemary is of particular importance for medical applications due to the presence of medicinal compounds such as (α-pinene, α-terpinene, camphene, and l,8-cineol)^16^. These compounds are widely used in the pharmaceutical and health industries. Polyamine compounds such as plant diamine putrescine, triamine spermidine, tetraamine, and spermine play essential roles in enhancing cellular defense against a wide range of abiotic stresses such as drought, salinity, and heavy metals^17^. These compounds are also regulators of plant growth and have a significant role at various levels of cell growth and development stages, gene expression, metabolism, etc.^18^. Spermine plays a vital function in protecting plants against different stress conditions. According to past reports, the amount of polyamine in plants increases significantly under stress conditions to protect plants from damage^19^. Moreover, these compounds can play a protective role and a hormone-like regulator role in the plant^20^.

Considering that the increase of medicinal compounds and secondary metabolites in medicinal plants occurs in the presence of environmental stresses such as drought, iron oxide nanoparticles and polyamines, on the other hand, help reduce the effects of drought stress, and their application increases the amount of secondary metabolites, particularly in medicinal plants. Therefore, the current research aimed to synthesize iron oxide nanoparticles by the green synthesis method from spinach aqueous extract and coat them with spermine, and also to examine the effect of these nanoparticles on the activity of antioxidant enzymes and secondary metabolites of *Rosmarinus officinalis* under drought stress conditions.

## Materials and methods

### Materials

2-Carboxyethyl 2-Pyridyl Disulfide, FeCl_3_. 6H_2_O, FeCl_2_. 4H_2_O, (3-Mercaptopropyl) trimethoxysilane 95% (MPTMS), Tetraethyl orthosilicate (TEOS), N-Hydroxysuccinimide 98% (NHS), 1-ethyl-3-(3-dimethylaminopropyl)-carbodiimide (EDC), and NaOH were purchased from Sigma-Aldrich (USA). Ammonia solution 25%, Aluminum chloride, Trichloroacetic acid, Thiobarbituric acid, Gallic acid, Polyvinylpyrrolidone (PVP) were purchased from Merck (Germany).

### Hydroalcoholic extract preparation

To prepare the hydroalcoholic extract, 10 g of spinach was first added to 100 ml of 80% methanol. The mixture was kept at room temperature for 24 under continuous stirring (50 rpm). After 24 hours, the obtained hydroalcoholic solution was centrifuged at 10,000 rpm for 10 minutes. The supernatant was isolated and filtered through a Whatman filter paper grade 2 to remove impurities.

### Iron oxide nanoparticle synthesis

Briefly, 3.33 g of iron (III) chloride hexahydrate (FeCl_3_. 6H_2_O) and 1.59 g of iron (II) chloride tetrahydrate (FeCl_2_. 4H_2_O) were dissolved with 100 ml of deionized. The solution was mixed under nitrogen gas at 80 °C for 10 min. Subsequently, 15 ml of hydroalcoholic spinach extract was added dropwise into the mixture under continuous stirring for 10 min. After five minutes, 60 ml of NaOH 1M was added to the solution. The reaction continued until the color of the solution changed from yellow to dark brown. Then the nanoparticles were collected using a magnet and then washed with deionized water to remove impurities.

### Fe_3_O_4_@SiO_2_-SS- SM nanoparticles synthesis

The Fe_3_O_4_@SiO_2_-SS-SM nanoparticle synthesis was performed using the method reported by Zhang et al. (2016)^21^. To synthesize the Fe_3_O_4_@SiO_2_-SH, 200 mg of Fe_3_O_4_ nanoparticles were dispersed in ethanol by sonication for 15 minutes, followed by sequential addition of 1 mL NH_3_.H_2_O, 1 mL MPTMS and 20 mL TEOS to the ultrasound water bath. Finally, the nanoparticles were separated using a magnet, washed five times with ethanol and water, and vacuum-dried overnight. The resulting Fe_3_O_4_@SiO_2_-SH was then conjugated with COOH with 200 mg of sulfhydrylated Fe_3_O_4_ nanoparticles dispersed in 12 mL methanol and were mixed with equal amounts of 2-carboxyethyl 2-pyridyl disulfide. The reaction was maintained for 36 hours, and the product was then washed and dried, as discussed earlier.

**Figure 1:**
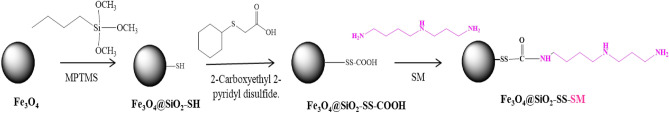
Schematic of Fe_3_O_4_-SM nanoparticle synthesis.

To synthesize the Fe_3_O_4_@SiO_2_-SS-SM nanoparticles, 200 mg of Fe_3_O_4_@SiO_2_-SS-COOH nanoparticle was dispersed in 16 mL PBS buffer (pH = 7.4). Then, NHS (10 mg) and EDC (20 mg) were added at room temperature for 1 hour to activate the carboxyl terminal for Fe_3_O_4_@SiO_2_-SS-COOH. After that, the mixed solution was added to 4 ml of PBS buffer that contained 200 mg of spermine and was stirred at 25 °C for two days. The final product of nanoparticles was washed several times with de-ionized water and ethanol and dried in a freeze drier. Figure 1, Shows a schematic of Fe_3_O_4_-SM nanoparticles synthesis.

### Rosmarinus officinalis genomic DNA extraction

Genomic DNA extraction from *Rosmarinus officinalis* was performed as described in the previous report^22^.

### Retardation assay

To interact DNA with Fe_3_O_4_-SM nanoparticles, a fixed amount of *Rosmarinus officinalis* DNA (5 µg) was combined with different concentrations of Fe_3_O_4_-SM nanoparticles (0, 0.25, 0.5, 1, 3, 4, and 5 mg) and then gently vortexed for 30 sec. The Fe_3_O_4_-SM/DNA complex was incubated at room temperature for 30 minutes. Finally, the interaction ability of nanoparticles with DNA was investigated by agarose gel electrophoresis. DNA was visualized using electrophoresis in 0.8% agarose gel at 80 V for 2 h, then stained with ethidium bromide and observed under UV light.

### DNA protection assays

To investigate the ability of Fe_3_O_4_-SM nanoparticles to protect DNA against *DNase I*, the Fe_3_O_4_-SM/DNA complex was prepared, as mentioned above. Then, the samples and the naked DNA (a fixed amount of 5 μg of DNA was considered for each sample) were incubated with *DNase I* (3 U ml−1 in 50 mM Tris-HCl) for 30 min at 37 °C. Next, the nucleases were inactivated by adding 5μL of 0.5 M EDTA solution (pH 8.0). Subsequently, the nanoparticles were disassembled from DNA by adding 1% (w/v) heparin and shaken for 4 h, at 37 °C^23^. The samples with naked DNA were analyzed using gel electrophoresis, as mentioned above. Moreover, to examine whether Fe_3_O_4_-SM nanoparticles can protect the DNA from ultrasound damage, the Fe_3_O_4_-SM/DNA complex and the naked DNA were sonicated in an ultrasonic (Sonorex Digitec, Bandelin, Germany) for 20 sec at 25 °C. Then, the nanoparticles were disassembled from DNA and analyzed using gel electrophoresis, as mentioned above.

### Cell suspension culture of Rosmarinus officinalis

Callus was first obtained by culturing *Rosmarinus officinalis* nodes on MS-Agar medium supplemented with 1 mg/l 2, 4-D, and 0.5 mg/L Kin. To prepare the cell suspension, a suitable amount of callus was added to 30 ml of liquid MS solution, and the cultures were incubated under stirring (80 rpm) at 25 °C for 1 wk. Cell-containing culture media were sub-cultured once a week by the aforementioned culture medium.

To evaluate the toxicity of Fe_3_O_4_ and Fe_3_O_4_-SM nanoparticles on *Rosmarinus officinalis* plant cells, different concentrations of Fe_3_O_4_ and Fe_3_O_4_-SM nanoparticles (100, 200, 300, 400, and 500 µg) were added separately to one milliliter of culture medium containing the cell and stored under stirring (80 rpm) at 25 for 24 h. To determine the effect of nanoparticles on *Rosmarinus officinalis* cell viability, a suitable amount of the cells treated with the different concentrations of the nanoparticles was stained by trypan blue solution (0.4% w/v). Next, equivalent volumes of trypan blue solution and cell suspensions were mixed and kept at room temperature for 5 minutes. Then, ∼20 μl of the stained cells were placed on a hemocytometer and investigated under light microscopy. The living cells (yellow) and dead cells (blue) were counted for all samples separately, and the percentage of cell viability in each sample was calculated based on the following equation Eq. (1):

1 $$\mathrm{Cell\,viability\,for\,each\,sample}\,\left(\mathrm{\%}\right)=\frac{\mathrm{The\,amount\,of\,living\,cells}}{\mathrm{Total\,amount\,of\,cells}}\times 100$$

Finally, the percentage of cell viability in each group was compared with the percentage of cell viability in control group cells based on the following equation (Equation 2):

2 $$\mathrm{Cell\,viability }\,(\mathrm{\%}) =\frac{\mathrm{Cell\,viability\,percentage\,for\,each\,sample}\times 100}{\mathrm{Cell\,viability\,percentage\,for\,control\,sample}}$$

### Application of Fe_3_O_4_and Fe_3_O_4_-SM nanoparticles on Rosmarinus officinalis

The effect of Fe_3_O_4_ and Fe_3_O_4_-SM nanoparticles application on biochemical and secondary metabolism properties of *Rosmarinus officinalis* was investigated under complete irrigation and drought stress. Experiments were performed in a completely randomized design (CRD) with three replications. The effect of Fe_3_O_4_ and Fe_3_O_4_-SM nanoparticles application on *Rosmarinus officinalis* was performed in different concentrations of nanoparticles (0, 50, and 100 mg/l). The nanoparticle application was carried out at the 5- to 6-leaf stage of *Rosmarinus officinalis.* Distilled water was used to treat the control sample.

### The effect of Fe_3_O_4_ and Fe_3_O_4_-SM on antioxidant enzyme activities and biochemical properties of Rosmarinus officinalis

The following methods were used to investigate the effect of Fe_3_O_4_ and Fe_3_O_4_-SM on antioxidant enzyme activities and biochemical properties of *Rosmarinus officinalis* (Table 1).

**Table 1.** The effect of Fe_3_O_4_ and Fe_3_O_4_-SM nanoparticles on antioxidant enzyme activities and biochemical properties of *Rosmarinus officinalis.*

Name	Method	Reference	Name	Method	Reference
Protein	Bradford, 1976	^24^	IC 50 of DPPH assay	Blois, 1958	^25^
Proline	Bates et al. 1973	^26^	Catalase	Aebi, 1983	^27^
Soluble sugar	Yemm and Willis, 1954	^28^	ascorbate- peroxidase	Nakano and Asada, 1981	^29^
Total phenol	Slinkard and Singleton, 1977	^30^	polyphenol oxidase	Raymond et al. 1993	^31^
Flavonoids	Chiu et al., 2002	^32^	H_2_O_2_	Patterson et al. 1984	^33^
Anthocyanin	Wanger, 1979	^34^	–	–	–

### Estimation of the terpenes content of Rosmarinus officinalis leaves using high-performance liquid chromatography (HPLC)

High-performance liquid chromatography was used to investigate the effect of Fe_3_O_4_ and Fe_3_O_4_ -SM nanoparticles on the Terpenes content of *Rosmarinus officinalis*. Briefly, one g of powder from leaves of *Rosmarinus officinalis* was extracted several times with 20 mL methanol. Next, the extracts were filtered and concentrated using a vacuum incubator. 20 µl of each extract was applied on HPLC (KNAUER-Germany). The stationary phase was an L10 column (Nitrile), and the flow rate was 0.5 mL/min^35^. UV detection (K2500) was used at λ = 275 nm for α-pinene and 315 nm for camphene ،α-terpinene and 1,8-cineol. 1 mg/ml of pure α-pinene, camphene, α-terpinene, and 1,8-cineol were used separately as HPLC standards. The elution solvent was composed of water containing 0.2 sulfuric acid (Solvent A) and methanol containing 0.2 sulfuric acid (Solvent B). Each Terpenes compound was quantified using a calibration curve prepared with each standard (Sigma-Aldrich, USA) and a co-chromatogram of the standards and samples. The experiments were repeated thrice, and each sample was assayed in triplicate^36,37^.

### Statistical analysis of the data

Each treatment was conducted with three replicates and comparisons concerning treatment tools were made by recruiting the least significant difference (LSD) at the 0.05 and 0.01 probability levels by SAS 9.4 software and Excel application software. The standard deviation of means ± was then calculated from the average of each treatment.

## Results and discussion

### Synthesis of Fe_3_O_4_ and Fe_3_O_4_-SM nanoparticles

Thermogravimetric analysis (TGA), UV-Vis spectrum, Fourier-transform infrared spectroscopy (FT-IR), and X-ray powder diffraction (XRD) were used to confirm the synthesis of Fe_3_O_4_ and Fe_3_O_4_-SM nanoparticles.

**Figure 2:**
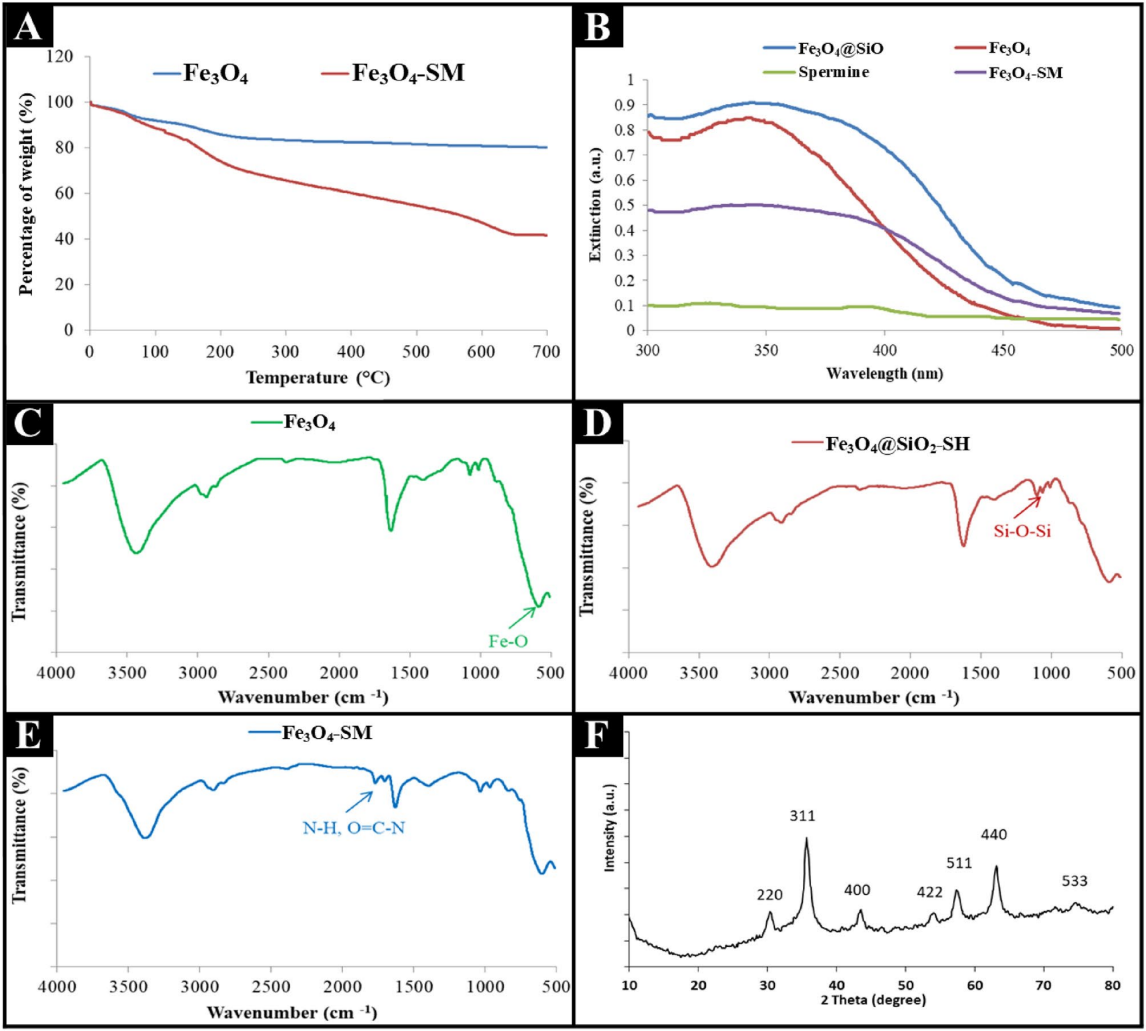
TGA curves of Fe_3_O_4_ and Fe_3_O_4_-SM nanoparticles (**A**); UV-Vis curves of different nanoparticles (**B**); FTIR spectra of Fe_3_O_4_ (**C**), Fe_3_O_4_@Sio_2_-SH (**D**), and Fe_3_O_4_-SM (**E**) nanoparticles; and XRD pattern of synthesized iron oxide nanoparticles (**F**).

The TGA curves obtained for Fe_3_O_4_ and Fe_3_O_4_-SM nanoparticles are shown in Figure 2A. The TGA analysis for the Fe_3_O_4_ nanoparticles showed two-step degradation. The initial mass loss of Fe_3_O_4_ in the range of 50 to 100 °C may be due to the loss of adsorbed water molecules from the nanoparticles. Moreover, the second stage of Fe_3_O_4_ nanoparticle degradation occurs between 100 and 300 °C, which may be some of the unstable compounds in the structure of Fe_3_O_4_ nanoparticles. The TGA analysis for the Fe_3_O_4_ nanoparticles demonstrated two-step degradation, while the Fe_3_O_4_-SM nanoparticles exhibit several steps of degradation in TGA analysis. This indicates that there are more compounds in the structure of Fe_3_O_4_-SM compared to Fe_3_O_4_ nanoparticles. As shown in Figure 2A, the trend in weight loss is similar for both Fe_3_O_4_ and Fe_3_O_4_-SM nanoparticles in the range of 50 to 100 °C. The Fe_3_O_4_-SM lost a significant amount of its remaining weight when the temperature reached 600 °C. Therefore, the temperature resistance for the Fe_3_O_4_-SM decreased significantly after Fe_3_O_4_ was modified with SM, which indicates successful conjugation of Fe_3_O_4_ and SM (Figure 2A).

The UV/Vis curve of Fe_3_O_4_ nanoparticles synthesized with spinach extract is reported in Figure 2B. As shown in Figure 2B, a strong peak in the range of 350–400 nm confirms that Fe_3_O_4_ is synthesized and stable. In addition, the absorption peak confirms the synthesis of Fe_3_O_4_ in nanometric dimensions. The UV/Vis results indicate no visible peak for spermine, and this result was consistent with the results of previous reports^38^. Meanwhile, the UV/Vis spectra showed that the peak of Fe_3_O_4_@SiO at 350-400 nm sharply decreased because of the spermine binding on the surface of the Fe_3_O_4_@SiO nanoparticle (Figure 2B).

### Fourier-transform infrared spectroscopy (FT-IR)

FT-IR technique was used to confirm the synthesis of nanoparticles and the presence of possible functional groups on the iron oxide nanoparticles synthesized with spinach extract (Figure 2C). The peaks at 575 cm^−1^ indicate the tensile vibration of the octahedral Fe–O structure, and the peak at 2931 cm^−1^ is related to the C–H bond tensile vibration. The peak at 575 cm^−1^ confirms the presence of the Fe_3_O_4_ compound and proves that no other compound of iron e.g., goethite or hematite, has been synthesized besides this one. The FT-IR spectra for the Fe_3_O_4_@SiO_2_–SH and Fe_3_O_4_@SiO_2_–SS-SM are illustrated in Figure 2D. As can be seen, the Fe_3_O_4_@SiO_2_–SH presented peak at 11050 cm^−1^ is related to the Si–O bond of Fe_3_O_4_@SiO_2_–SH nanoparticles.

The peaks at 1645–1755 cm^−1^ in the FT-IR spectra for the Fe_3_O_4_@SiO_2_-SS–SM confirmed the conjugation of SM to the Fe_3_O_4_@SiO_2_-SS–COOH. Once the SM is conjugated with the Fe_3_O_4_@SiO_2_-SS–COOH, the SM peaks from 1645 to 1755 cm^−1^, which can be attributed to the amide and amide amino groups will indicate the attachment of SM to the surface of Fe_3_O_4_@SiO_2_-SS–COOH nanoparticles (Figure 2E)^39,40^.

### X-ray powder diffraction (XRD)

XRD technique was used to investigate the crystalline structure of the synthesized iron oxide nanoparticles. Comparison of Miller indices of XRD peak values, i.e., 220, 311, 400, 511, and 440 that respectively correspond to the angles 30.9°, 35.9°, 43.8°, 57.7°, and 62.9° in the synthesized sample shown in Figure 2F and the standard spectrum in the Joint Committee on Powder Diffraction Standards (JCPDS) code 19-0629 indicates the accuracy of magnetite nanoparticle synthesis^41,42^.

The broadening of the XRD peaks indicates the small size of the synthetic magnetite. The size of the synthesized nanomagnetite crystals was calculated using the characteristic peaks through the Debye-Scherer equation. The average particle size was calculated to be about 18 nm at the main peak locations. This pattern also confirmed the cubic structure of synthetic nanoparticles.

### The images of transmission electron microscopy (TEM) and scanning electron microscopy (SEM)

**Figure 3:**
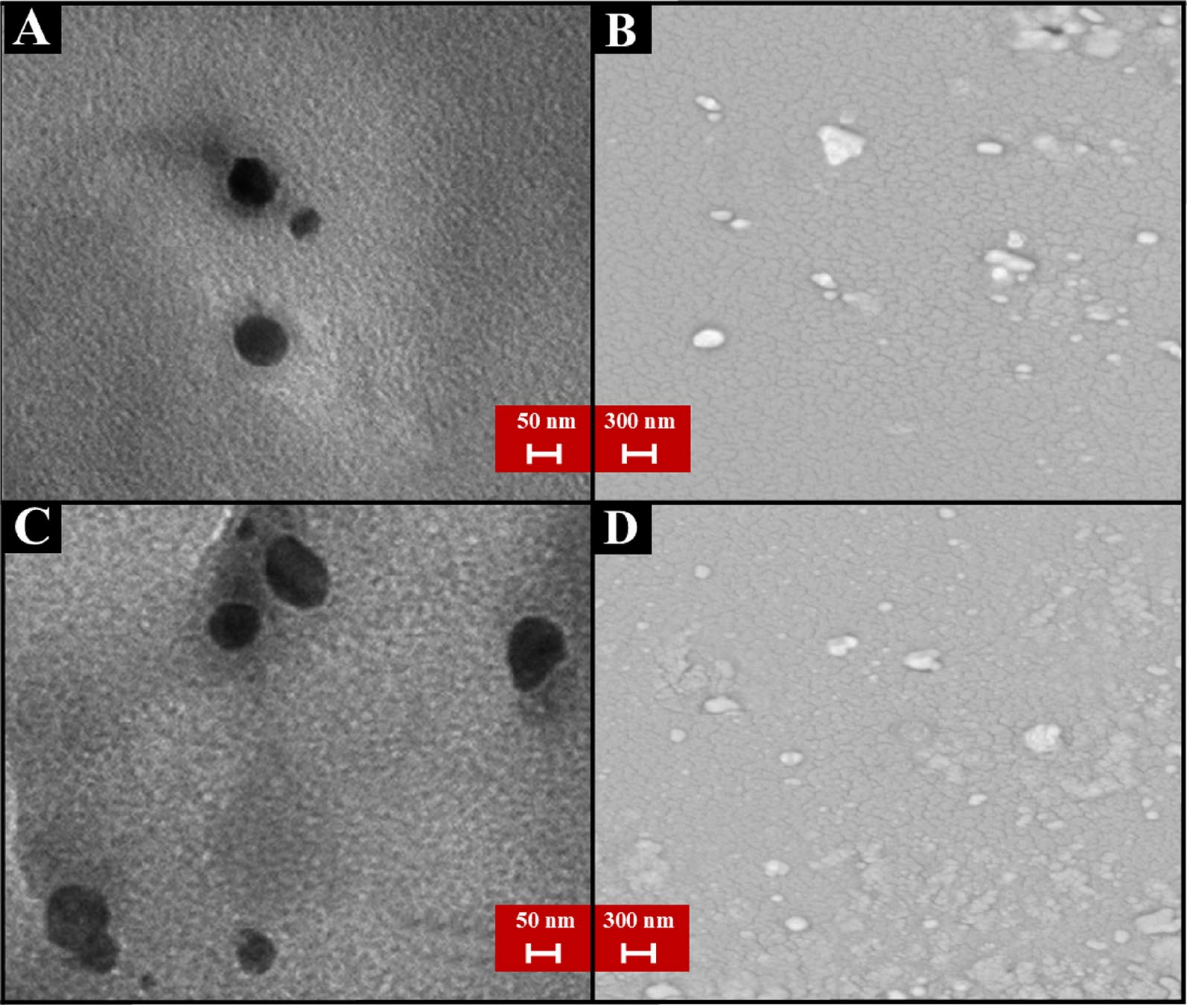
TEM images of the Fe_3_O_4_ (**A**), SEM images of the Fe_3_O_4_ (**B**), TEM images of the Fe_3_O_4_-SM nanoparticles (**C**), and SEM images of the Fe_3_O_4_-SM nanoparticles (**D**).

The TEM images of the Fe_3_O_4_ and Fe_3_O_4_-SM nanoparticles showed that the obtained nanoparticles were about 10–40 nm in size, which was congruent with the results of DLS. The nanoparticles also had a spherical structure consistent with the results of previous research. After coating the iron oxide nanoparticles with spermidine, the particle size increased slightly, and in addition to the spherical structure, oval structures were observed (Figure 3). The morphology of nanoparticles has a significant effect on their properties. According to past research, spherical nanoparticles have a higher transfer rate to cells than other nanoparticles' morphologies^43,44^. Also, SEM images of the synthesized Fe_3_O_4_ and Fe_3_O_4_-SM nanoparticles are shown in Figure 3B,D. This shape is used to confirm the size of the nanoparticles. SEM provided further insight into the surface morphology of the Fe_3_O_4_ and Fe_3_O_4_-SM. The experimental results showed that the diameter of the prepared Fe_3_O_4_ and Fe_3_O_4_-SM, as measured by the SEM images at 300 nm magnification, was approximately 10-40 nm, and the shape was found to be spherical, as shown in Figures 3B,D. The above results are in agreement with the findings of the research^45^.

### The dynamic light scattering (DLS) results of samples

**Figure 4:**
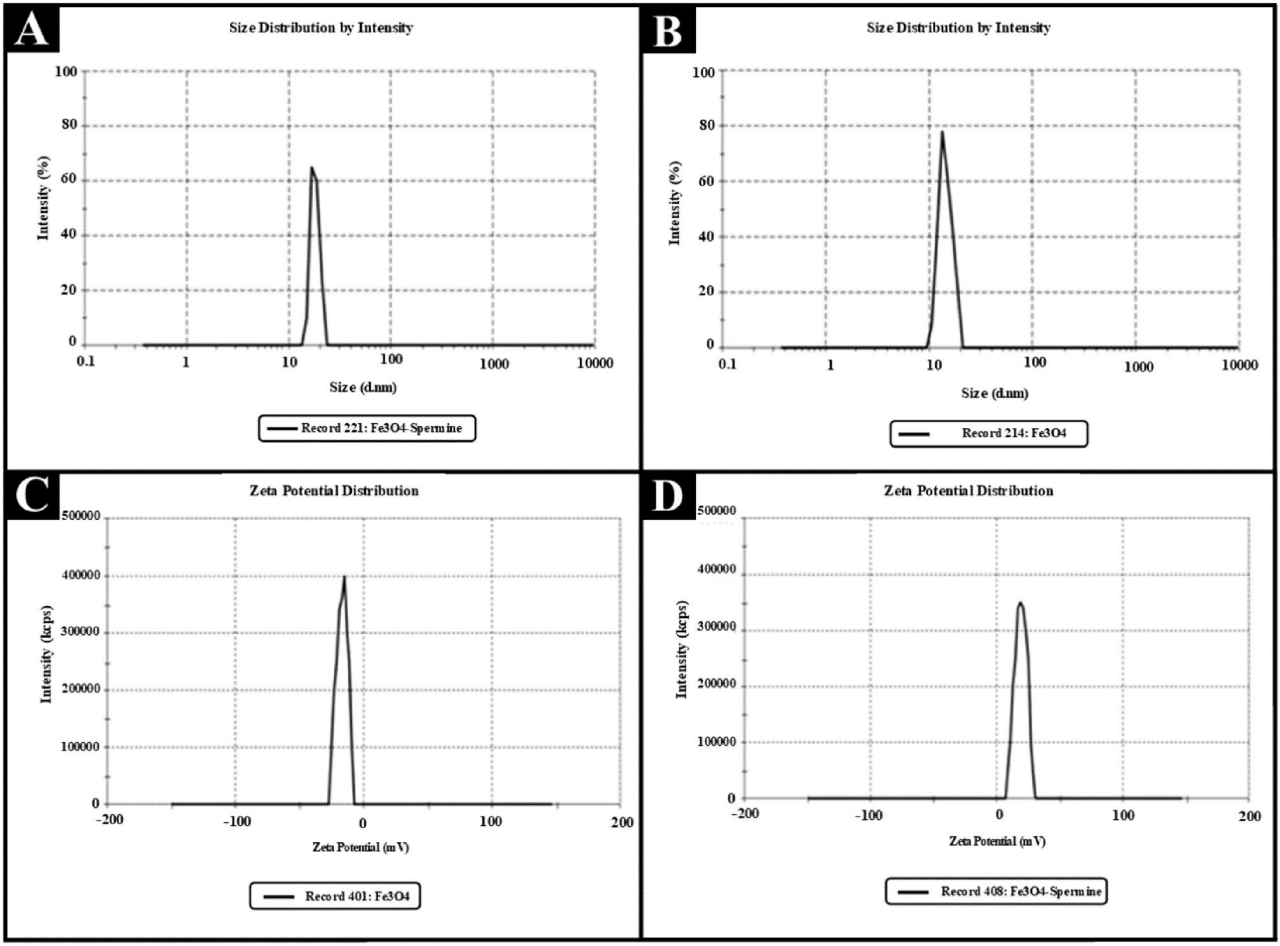
DLS results of Fe_3_O_4_ and Fe_3_O_4_-SM nanoparticles (**A** and **B**) the zeta potential of the Fe_3_O_4_-SM and Fe_3_O_4_ nanoparticles respectively; (**C** and **D**) the particles size of Fe_3_O_4_ and Fe_3_O_4_-SM nanoparticles respectively.

The results of DLS showed that the size of Fe_3_O_4_ nanoparticles was about 17 ± 3 nm. By coating the nanoparticles with spermine, their size reached 23 ± 16 nm. Interestingly, the surface charge of Fe_3_O_4_ nanoparticles increased significantly after spermine coating. Based on these results, the surface charge of the Fe_3_O_4_ nanoparticles was −3.2 ± 0.35 mV, while after coating by spermine, the surface charge increased to 18.42 ± 3.2 mV. The cationic charge of the amine groups in spermine is one of the reasons for the increase in the surface charge of these nanoparticles (Figure 4). The absorption of nanoparticles by cells increases significantly with decreasing nanoparticle size. Therefore, the synthesis of small nanoparticles is essential for their uptake by the cells. Since Fe_3_O_4_ and Fe_3_O_4_-SM nanoparticles had a spherical structure and an average particle size of less than 20 nm, these nanoparticles have a high ability to be absorbed by plant cells.

### Magnetic property determination

**Figure 5:**
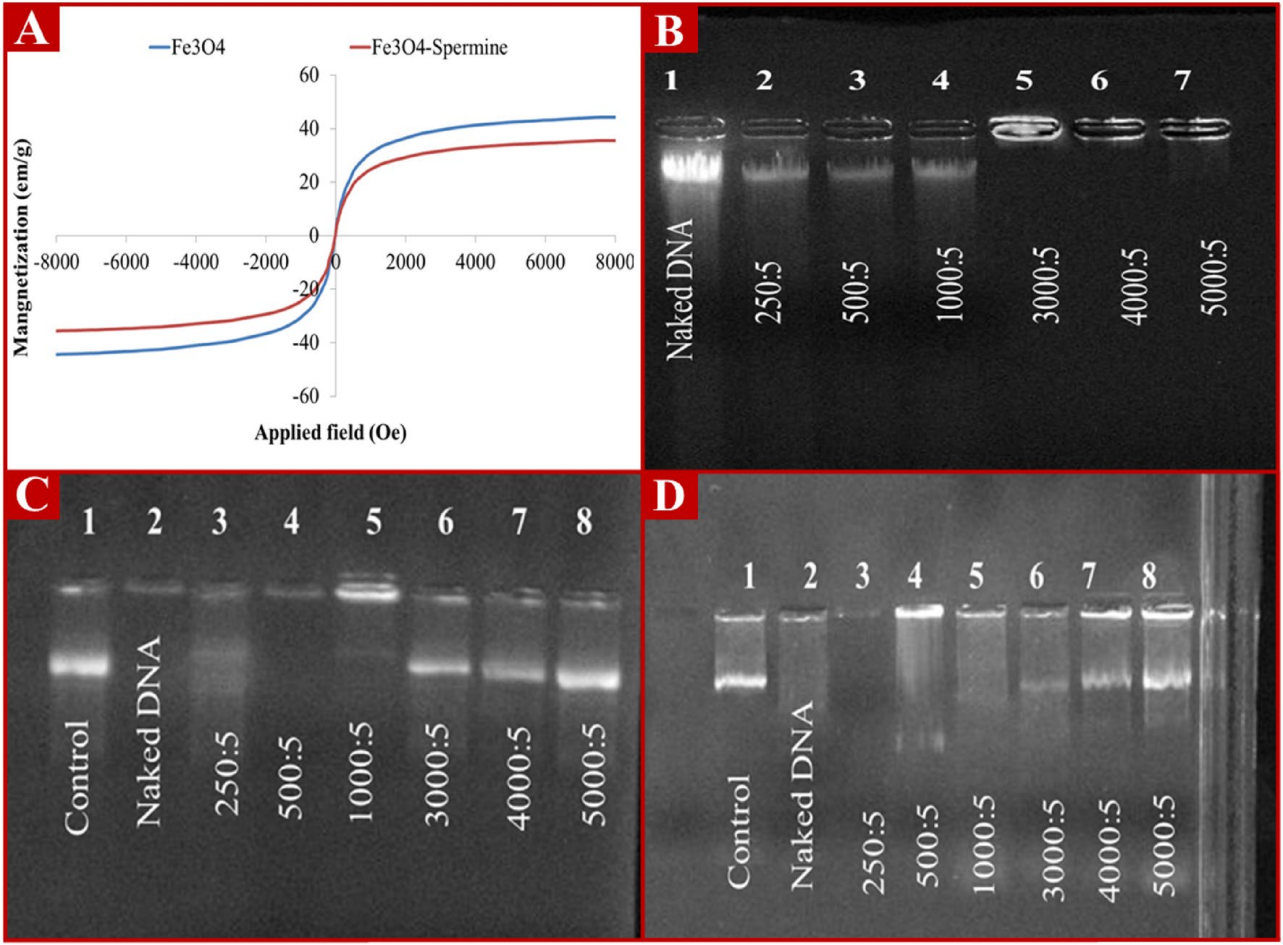
Magnetic behavior of Fe_3_O_4_ and Fe_3_O_4_-SM nanoparticles (**A**), Image of the agarose gel from the naked DNA (lane 1) and Fe_3_O_4_-SM/DNA complex prepared at different ratios of Fe_3_O_4_-SM nanoparticles to DNA (lanes 2 to 7) (**B**), Agarose gel images of Fe_3_O_4_-SM nanoparticles capability in protecting DNA against enzymatic degradation (**C**) and ultrasonic waves (**D**). The first well (DNA without treatment with enzymes and ultrasonic waves), wells 2 to 8, were respectively treated with the Fe_3_O_4_-SM/DNA complex prepared in ratios zero (DNA without Fe_3_O_4_-SM nanoparticles coating), 250, 500, 1000, 3000, 4000 and 5000 µg of Fe3O4-SM nanoparticles to 5 µg of DNA.

The saturation magnetization was 41.3 emu/g for Fe_3_O_4_ nanoparticles (Figure 5A). These data are in good agreement with a previous report^46^. The saturation magnetization values of Fe_3_O_4_ nanoparticles significantly decreased from 41.3 to 32.7 emu/g after being coated with spermine (Fe_3_O_4_-SM). This reduction in saturation magnetization is often observed in nanoparticles, with encapsulation of the magnetic nanoparticles into biodegradable nanoparticles^47^.

### Examining the ability of Fe_3_O_4_-SM nanoparticles to interact and protect DNA

The present study results showed that the amine groups in spermine can interact electrostatically with the negative charge of the phosphate group in DNA due to their cationic charge. As shown in Figure 5B, there was no significant difference in the DNA banding pattern between the naked DNA and the DNA/Fe_3_O_4_-SM nanoparticles were prepared at the mass ratios lower than that of 1000 of the nanoparticles to 1 of the DNA (% w/w). However, no DNA bands were observed in the agarose gel in the mass ratio of 3000 Fe_3_O_4_-SM nanoparticles to 5 of DNA (% w/w) and above. These results show that by neutralizing the negative charge of DNA by Fe_3_O_4_-SM nanoparticles, the migration toward the positive polar of agarose gel is completely stopped and remains in the well (Figure 5B). As shown in Figure 5C; there was no visible band of DNA detected in the agarose gel after the treatment of the naked DNA by ultrasound and *DNase Ι*. This may be due to the breakdown of DNA by ultrasound and it's being removed from the agarose gel during electrophoresis. A similar trend was observed for the Fe_3_O_4_-SM/DNA complex prepared at a mass ratio of less than 3,000 µg of Fe_3_O_4_-SM nanoparticles to 5 µg of DNA.

With the increase in the ratio of Fe3O4-SM/DNA nanoparticles to over 3000 µg, DNA bands were observed in agarose gel. Observations of the DNA band show that nanoparticles can protect DNA from enzymatic digestion and ultrasound (Figure 5D). DNA-coated nanoparticles appear to protect DNA from ultrasonic waves like a shield. Also, the binding of nanoparticles to DNA causes it to be coated, thus preventing the binding of restriction enzymes to DNA^23^.

### Biocompatibility of Fe_3_O_4_ and Fe_3_O_4_ -SM nanoparticles on Rosmarinus officinalis cells

**Figure 6:**
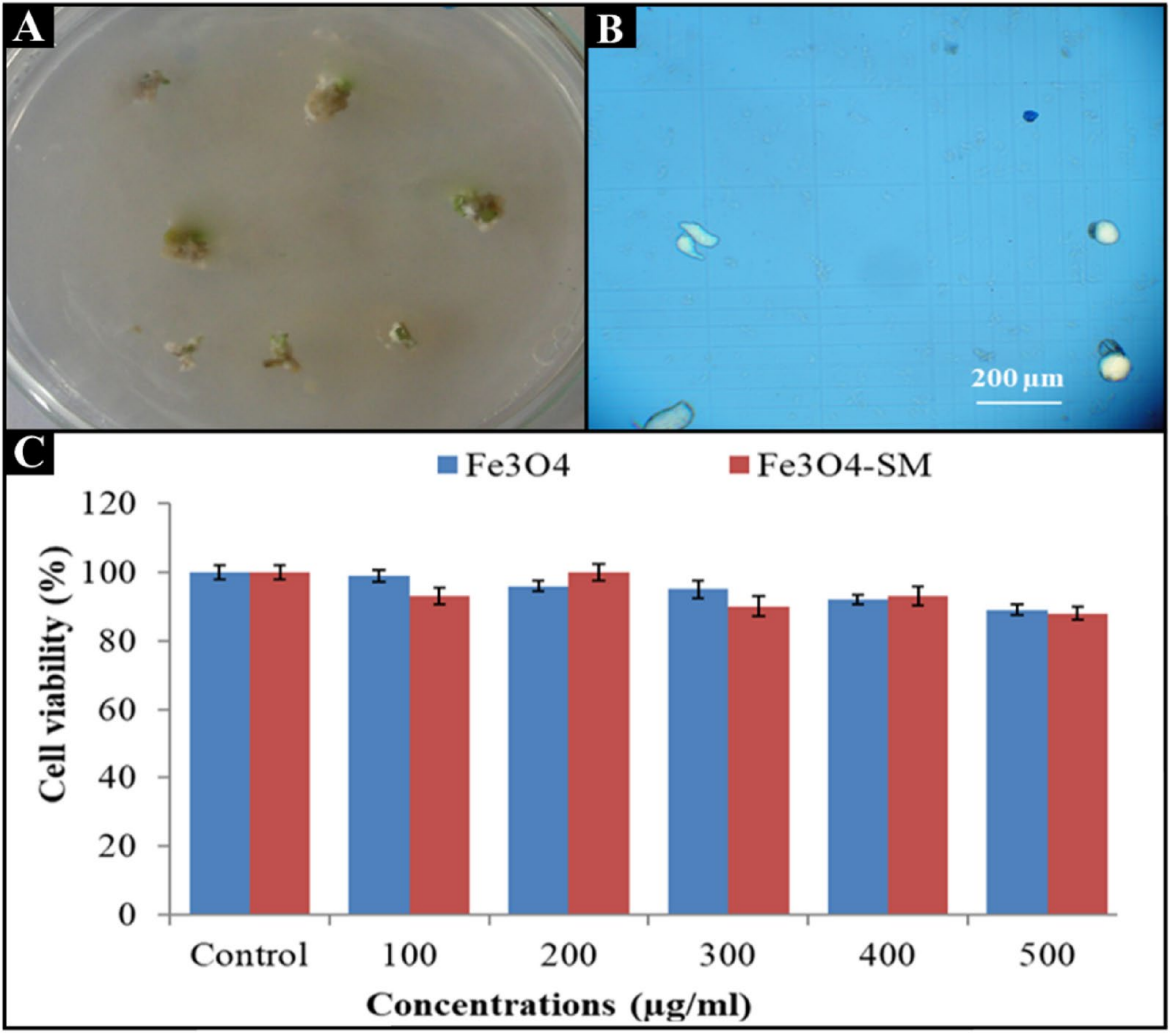
Callus image of the *Rosmarinus officinalis* plant (**A**) plant cell microscope image of *Rosmarinus officinalis* treated with Fe_3_O_4_ -SM nanoparticles at the concentration of 1 mg/ml after Trypan blue staining (**B**) Comparison of the mean effect of Fe_3_O_4_ -SM nanoparticles on the viability of *Rosmarinus officinalis* cells (**C**).

The effect of Fe3O4 and Fe_3_O_4_-SM nanoparticles on *Rosmarinus officinalis* cells showed that the minimum viability rate of *Rosmarinus officinalis* cells treated with different concentrations of Fe_3_O_4_ and Fe_3_O_4_-SM nanoparticles compared to the control group was 88%. After treatment, *Rosmarinus officinalis* cells were observed with 1 mg/ml of Fe_3_O_4_ nanoparticles. These results indicate that Fe_3_O_4_ and Fe_3_O_4_-SM nanoparticles have good biocompatibility with *Rosmarinus officinalis* cells (Figure 6).

### The effect of drought stress and Fe_3_O_4_-SM application on biochemical parameters of Rosmarinus officinalis

Based on variance analysis, the interaction effects of nanoparticles (Fe_3_O_4_ and Fe_3_O_4_-SM) and drought stress were significant in the biochemical parameters of *Rosmarinus officinalis* (Table 2).

**Table 2:** Analysis of variance of biochemical traits of *Rosmarinus officinalis* under the influence of nanoparticles and drought stress.

S.O.V	D.F	Mean of squares						
		Proline	Soluble sugar	Total phenol	Flavonoids	Anthocyanin	IC 50 of DPPH assay	Protein
Nanoparticles (NPs)	4	0.037^**^	0.564^**^	0.422^**^	115.64^**^	1214.06^**^	448.35^**^	0.010^**^
Drought stress (DS)	1	0.053^**^	1.771^**^	0.856^**^	494.10^**^	2126.89^**^	4.21^**^	0.057^**^
NPs* DS	4	0.020^**^	0.144^**^	0.198^**^	50.62^**^	341.60^**^	71.97^**^	0.006^**^
Error	20	0.0003	0.0005	0.018	0.41	10.88	0.584	0.001
C.V (%)	-	4.57	1.63	9.82	2.97	6.81	0.61	9.65

^**^ indicating significant at 1 % level, respectively.

Results showed that the soluble sugar and proline content of *Rosmarinus officinalis* increased significantly under drought stress. The results also showed that these amounts even increased after applying iron oxide and spermine-iron oxide nanoparticles to *Rosmarinus officinalis*. In fact, among the treatments, the application of 50 mg/L of Fe_3_O_4_-SM in drought stress (FS_50_I_1_) led to an increase in proline and soluble sugars, compared to the control treatment (Table 3). Also, similar trends were observed for anthocyanin content. As shown in Table 3, the highest anthocyanin content was observed with treatment with 100 mg/L of Fe_3_O_4_-SM under drought stress conditions (FS_100_I_1_) (80.4±7.5 µM/g FW). In other words, anthocyanins are part of phenolic compounds and form a large group of secondary metabolites; therefore, while having antioxidant properties, they act as free radical receptors and protect plants against oxidative stress^48^. Considering that the highest amount of anthocyanin was obtained under drought-stress conditions, the explanation for this rise can be linked to the photoprotective action of anthocyanin through the elimination of reactive oxygen species during oxidative stress. Plants are a rich source of phenolic compounds, which are the most important natural and secondary antioxidants. Therefore, among the secondary metabolites, we can mention the phenolic-flavonoid compound, which increases under conditions of oxidative stress. As a result, the highest amounts of total phenol (1.9±0.14 mg/g FW) and flavonoid content (41.1±4.8 mg/g FW) were observed in the applications of 100 mg/L (FS_100_I_1_) and 50 mg/L of Fe_3_O_4_-SM in drought stress conditions (FS_50_I_1_), respectively. Plants appear to boost the production of secondary metabolites such as phenolic compounds and anthocyanin in response to drought stress to cope with the impacts of reactive oxygen species and adapt to new conditions. The researchers reported that drought stress leads to an increase in secondary metabolites, including anthocyanins, phenols, and flavonoids^49^, which is consistent with the present study. Furthermore, the effects of drought stress and spermine-iron oxide nanoparticle applications on *Rosmarinus officinalis* were investigated by measuring 1,1-diphenyl-2-picrylhydrazyl (DPPH) in a methanolic extract of *Rosmarinus officinalis*. The IC50 value is the amount of extract required to scavenge 50% of the DPPH radical. therefore, the decrease in the IC50 value of the extract may reflect its potent antioxidant properties. The lowest concentration required for scavenging 50% of the DPPH radical was observed in FS_100_I_0_ (110.51±9.5 µg/g FW). Therefore, it can be concluded that the application of Fe_3_O_4_-NPs by spermine improves plant stability against free radicals (Table 3).

**Table 3:** Means comparison of biochemical traits as affected by nanoparticles and drought stress

Treatments	Soluble sugar (mg/g FW)	Proline (mg/g FW)	Anthocyanin (µM/g FW)	Total phenol (mg/g FW)	Flavonoids (mg/g FW)	IC 50 of DPPH assay µg/g FW	Protein (mg/g FW)
F_0_I_0_	1.04±0.06	0.29±0.01	37.4±4.5	1.1±0.04	26.35±1.5	136.51±12.5	0.29±0.03
F_50_I_0_	0.95±0.05	0.32±0.02	35.1±6.7	0.98±0.07	23.4±2.5	127.15±11.6	0.31±0.02
F_100_I_0_	1.2±.0.09	0.44±0.02	32.7±3.7	1.4±0.09	18.5±1.3	133.15±13.1	0.36±0.04
FS_50_I_0_	1.32±0.08	0.44±0.03	42.9±3.3	1.02±0.08	26.2±2.4	116.84±10.6	0.41±0.01
FS_100_I_0_	1.43±0.11	0.29±0.01	52.7±6.2	1.5±0.12	25.6±2.7	110.51±9.5	0.43±0.03
F_0_I_1_	1.5±0.07	0.31±0.02	41.4±3.1	1.3±0.08	33.1±2.9	129.8±8.4	0.28±0.01
F_50_I_1_	0.98±0.03	0.38±0.04	38.6±2.7	1.35±0.06	22.6±1.7	122.85±9.7	0.29±0.01
F_100_I_1_	1.9±0.1	0.39±0.04	51.7±5.4	1.2±0.08	30.7±3.8	133.6±11.8	0.29±0.05
FS_50_I_1_	2.1±0.13	0.58±0.08	73.9±6.8	1.7±0.12	41.1±4.8	118.6±10.5	0.3±0.02
FS_100_I_1_	1.82±0.09	0.52±0.05	80.4±7.5	1.9±0.14	32.9±2.8	121.5±13.4	0.32±0.03

F_0_, F_50,_ and F_100_: Iron oxide levels (0, 50 and 100 mg/L respectively), FS_0_, FS_50_ and FS_100_: spermine-iron oxide levels (0, 50 and 100 mg/L respectively), I_0_ and I_1_: Irrigation levels (complete irrigation and drought stress respectively).

On the other hand, the analysis of variance showed that the effects of nanoparticles and drought stress significantly affected protein content (Table 2). As can be seen in Table 3, the highest content of protein was (0.43±0.03 mg/g FW) that was obtained from 100 mg/L Fe_3_O_4_-SM (FS_100_I_0_) in complete irrigation conditions, and the lowest content of protein was in no application of nanoparticles in drought stress conditions (0.28±0.01 mg/g FW). The absence of nanoparticles and the drought stress condition reduced the content of protein by approximately 53.57% when compared to the application of 100 mg/L Fe_3_O_4_-SM in complete irrigation conditions. The decrease in protein content under drought stress appears to be caused by the reaction of protein with free radicals, which results in amino acid changes, a decrease in protein synthesis, an accumulation of free amino acids, including proline, and an increase in the activity of protein degrading enzymes. Furthermore, when 100 mg/L Fe_3_O_4_-SM was used, the soluble proteins increased significantly. It seems that the increase of soluble proteins in the application of Fe_3_O_4_-NP coating with spermine is due to the synthesis of new proteins, the increase in the level of proteins related to stress tolerance, such as proline, or the role of this nanoparticle in dealing with oxidative stress. Also, The effect of Fe_3_O_4_-NP and spermine in preventing the structural and functional destruction of the cell membrane, increasing the stability of lipids in the cell membrane of crop plants exposed to drought and heat stress has been reported by other researchers^50^.

### The effect of drought stress and spermine coated iron nanoparticles on hydrogen peroxide content, antioxidant enzyme activity, and secondary metabolites of Rosmarinus officinalis

Based on variance analysis, interaction effects of nanoparticles (Fe_3_O_4_ and Fe_3_O_4_-SM) and drought stress were significant on hydrogen peroxide content, antioxidant enzyme activity, and secondary metabolites of *Rosmarinus officinalis* (Table 4).

**Table 4:** Analysis of variance of H_2_O_2_ content, antioxidant enzyme activity, and secondary metabolites of *Rosmarinus officinalis* under the influence of nanoparticles and drought stress

S.O.V	D.F	Mean of squares							
		H_2_O_2_	CAT	APX	PPO	α-pinene	camphene	l,8-cineol	α-terpinene
Nanoparticles (NPs)	4	0.323^**^	17.37^**^	27166.5^**^	25993.4^**^	2.40^ns^	0.250^**^	15.92^**^	73.40^**^
Drought stress (DS)	1	3.07^**^	44.38^**^	95466.0^**^	70488.9^**^	89.96^**^	0.453^**^	0.302^**^	966.84^**^
NPs* DS	4	0.199^**^	8.57^**^	11702.8^**^	2466.4^*^	16.57^**^	1.64^**^	1.83^**^	84.68^**^
Error	20	0.033	1.67	146.73	808.70	1.39	0.062	0.032	3.17
C.V (%)	–	7.65	10.65	3.73	5.33	10.25	10.55	4.45	5.86

ns, * and ** indicating non-significant and significant at 5 and 1 % level, respectively.

**Figure 7:**
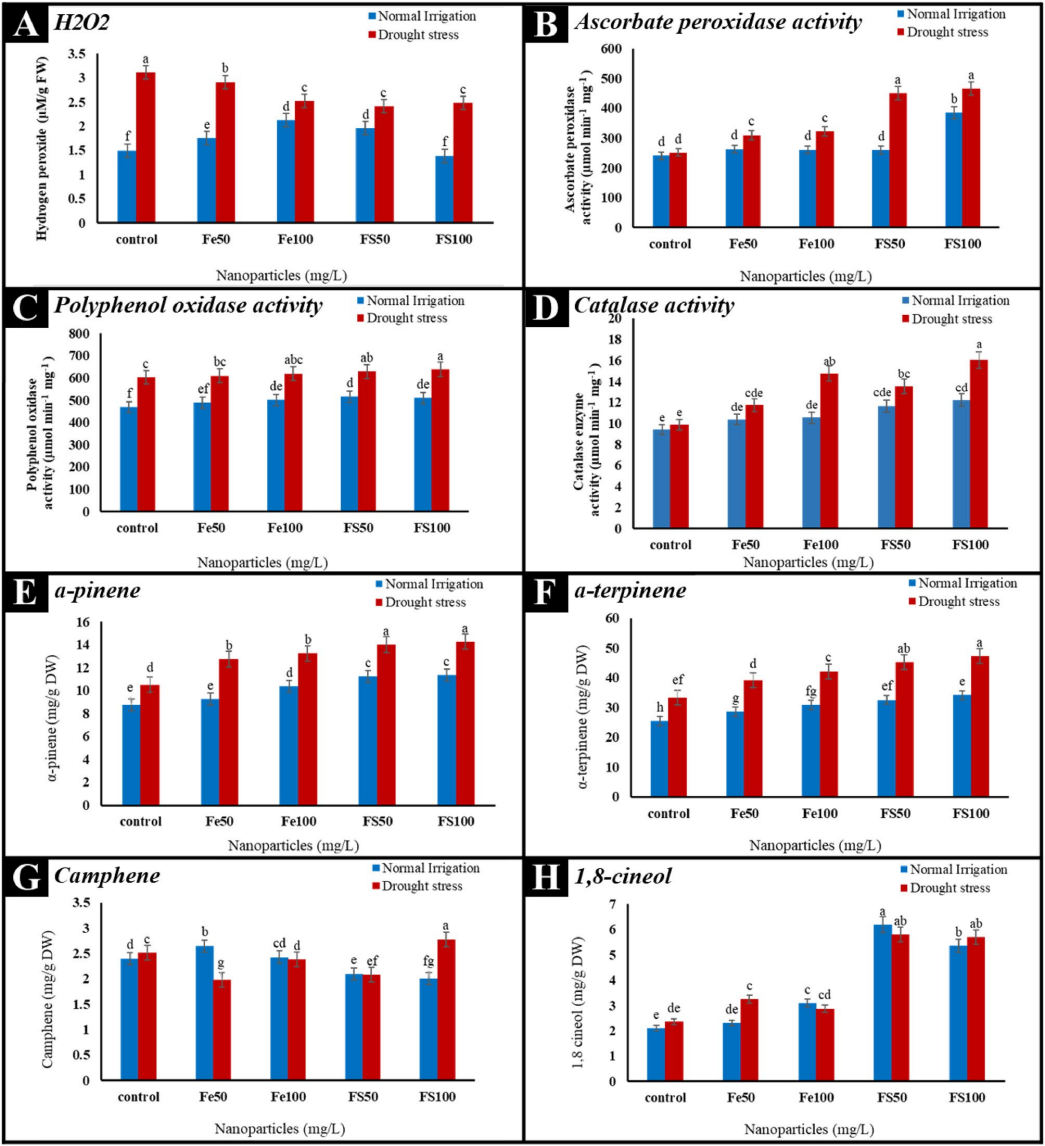
The effect of Fe_3_O_4_ and Fe_3_O_4_-SM nanoparticles application on hydrogen peroxide content and antioxidant enzyme activity of *Rosmarinus officinalis* (**A**, **B**, **C**, **D**), The effect of Fe_3_O_4_ and Fe_3_O_4_-SM nanoparticles on secondary metabolites of *Rosmarinus officinalis* under normal irrigation and drought stress (**E**, **F**, **G**, **H**).

As shown in Figure 7A, the application of Fe_3_O_4_-NPs and Fe_3_O_4_ coating by spermine had significant effect on the hydrogen peroxide content (H_2_O_2_) of *Rosmarinus officinalis*. Drought stress significantly increased the hydrogen peroxide content in *Rosmarinus officinalis.* The increase in levels of H_2_O_2_ content under drought stress has also been reported in previous studies^51,52^. This increase depends on the severity of the drought stress and the intensity of cell membrane damage. Under drought stress conditions, the application of Fe_3_O_4_-SM to *Rosmarinus officinalis* significantly reduced H_2_O_2_ content. It seems that part of the decrease in H_2_O_2_ content in the case of the application of Fe_3_O_4_-SM is related to the increase in the activity of some important enzymes of the oxidative defense system, such as catalas (Figure 7D). It has been reported that the use of Fe_3_O_4_-NPs and spermine increases the activity of some important enzymes participating in the oxidative defense system, such as catalase and peroxidase, and decreases H_2_O_2_ in plants that are under drought stress^53^.

Also, it has been reported that H_2_O_2_, as a regulatory factor, plays an important role in the activation of genes encoding proteins that are involved in the defense against oxidative stress^54^. Also, it has been reported that under conditions of drought stress, the content of hydrogen peroxide increases, and antioxidant enzymes are the most important compounds in deactivating free radicals^55^. The antioxidant role of polyamines has already been established. Polyamines decrease the levels of reactive oxygen species (ROS) in cells by increasing antioxidant enzyme activity^56^. It has been reported that the use of polyamines, including spermine, leads to the reduction of H_2_O_2_ in conditions of environmental stress^57^.

Our results showed that drought stress significantly increased the activity of antioxidant enzymes. In addition, it was found that coating Fe_3_O_4_ nanoparticles with spermine significantly increased the activity of antioxidant enzymes compared to Fe_3_O_4_ nanoparticles. So, among the treatments, the highest activities of ascorbate peroxidase, polyphenol oxidase, and catalase enzymes in *Rosmarinus officinalis* were obtained under drought stress conditions (Figure 7B–D). Indeed, environmental stress affects the plant's activity. The current study demonstrates that an increase in antioxidant enzyme activity is one of the important mechanisms that occurs when the plant experiences environmental stresses, such as drought stress, to increase the plant's tolerance to these conditions. An increase in the activity of antioxidant enzymes such as catalase ascorbate and peroxidase can be considered a cellular defense mechanism against oxidative damage under stress conditions^58^. Also, the results showed that the use of Fe_3_O_4_ coated with spermine led to an increase in the activity of these antioxidant enzymes compared to no application of nanoparticles. In other words, the application of 100 mg/L of Fe_3_O_4_ coated with spermine led to an increase in the ascorbate peroxidase (466 μmol min^−1^ mg^−1^), polyphenol oxidase (639 μmol min^-1^ mg^−1^), and catalase (16.03 μmol min^−1^ mg^−1^) enzymes in *Rosmarinus officinalis* under drought stress conditions (Figure 7B–D). In other words, the application of 100 mg/L of Fe_3_O_4_ coated with spermine led to an increase of 36.35%, 70.35%, and 93.36% respectively in polyphenol oxidase, catalase, and ascorbate peroxidase compared to the control treatment. One of the reasons for the increased activity of antioxidant enzymes in drought-stress conditions can be due to the use of Fe_3_O_4_ coated with spermine compared to its non-use. Because Fe_3_O_4_-SM protects the cell membrane against lipid peroxidation, it makes the plant tolerate stress conditions and increases the activity of antioxidant enzymes such as catalase and ascorbate peroxidase.

Also, as shown in the mean comparison results the highest content of α-pinene (14.25 mg/g DW) and α-terpinene (49.29 mg/g DW) were observed in the application of 100 mg/L of Fe_3_O_4_ coated with spermine under drought stress (FS_100_I_1_) that showed a non-significant difference with FS_50_I_1_ treatment (Figure 7E,F). It has been reported that drought stress can increase the concentration of secondary metabolites and increase the expression of genes involved in the synthesis of these metabolites in medicinal plants^59^. Also, the results showed that under drought stress conditions, it leads to a significant increase of l,8-cineol, and camphene. On the other hand, the use of treatments FS_50_ and FS_100_ also had a positive effect on the increase of l,8-cineol, and camphene compared to the control treatment (Figure 7G,H). Generally, the results showed that drought stress increased the secondary metabolite content in *Rosmarinus officinalis*. Phenolic compound levels in *Rosmarinus officinalis* also increased after treatment with spermine-iron oxide nanoparticles. The results of our study are in agreement with previous studies. Previous reports have shown that under various stresses, plant energy is used to produce secondary metabolites to help to prevent cellular damage by free radicals^60^.

## Conclusion

Plants try to keep themselves in ideal conditions under stress. Therefore, many plant metabolites change quantitatively and qualitatively in this respect. What was observed in this study also confirms this fact. Different plant compounds show different responses to various stresses. The results of the present study showed that the effect of environmental stress on the increase of secondary metabolites in plants, and the expression of relevant genes in different tissues may be affected differently. The results showed that the effect of drought stress in increasing secondary metabolites such as l,8-cineol, camphene, and α-terpinene in *Rosmarinus officinalis* is intensified by the application of iron oxide nanoparticles and spermine-coated iron oxide nanoparticles. The results also showed that the simultaneous effects of iron oxide nanoparticles and spermine-coated iron oxide nanoparticles increased the activity of some antioxidant enzymes and secondary metabolites in *Rosmarinus officinalis*. Polyamine compounds, such as spermine, can enhance the pharmacological potential of this plant by further stimulating the biosynthesis of secondary metabolites, such as glycosides, flavonoids, phenols, etc. In general, it seems that the application of spermine-coated iron oxide nanoparticles can be a suitable method to reduce the effects of drought stress.

## Data Availability

The datasets generated during and/or analyzed during the current study are available from the corresponding author on reasonable request.
